# A consultation training program for physicians for communication about complementary medicine with breast cancer patients: a prospective, multi-center, cluster-randomized, mixed-method pilot study

**DOI:** 10.1186/s12885-016-2884-y

**Published:** 2016-11-04

**Authors:** Susanne Blödt, Nadine Mittring, Lena Schützler, Felix Fischer, Christine Holmberg, Markus Horneber, Adele Stapf, Claudia M. Witt

**Affiliations:** 1 Institute for Social Medicine, Epidemiology and Health Economics, Charité - Universitätsmedizin Berlin, Luisenstrasse 57, 10117 Berlin, Germany; 2 Department of Psychosomatic Medicine, Center for Internal Medicine and Dermatology, Charité - Universitätsmedizin Berlin, Berlin, Germany; 3 Institute of Public Health, Charité - Universitätsmedizin Berlin, Berlin, Germany; 4 Department of Internal Medicine, Division of Oncology and Hematology, Paracelsus Medical University, Klinikum Nuremberg, Nuremberg, Germany; 5 Institute for Complementary and Integrative Medicine, University of Zurich and University Hospital Zurich, Zurich, Switzerland

**Keywords:** Communication, Breast cancer, Complementary and integrative medicine

## Abstract

**Background:**

The aim was to develop and evaluate a training program for physicians for communicating with breast cancer patients about complementary medicine (CM).

**Methods:**

In a cluster-randomized pilot trial eight breast cancer centers (two physicians per center) were randomized to either a complementary communication training program (9 h e-learning + 20 h on-site skills training) or to a control group without training. Each physician was asked to consult ten patients for whom he or she is not the physician in charge. We used mixed methods: Quantitative outcomes included physicians’ assessments (empathy, complexity of consultation, knowledge transfer) and patients’ assessments (satisfaction, empathy, knowledge transfer). For qualitative analyses, 15 (eight in the training and seven in the control group) videotaped consultations were analyzed based on grounded theory, and separate focus groups with the physicians of both groups were conducted.

**Results:**

A total of 137 patients were included. Although cluster-randomized, physicians in the two groups differed. Those in the training group were younger (33.4 ± 8.9 vs. 40.0 ± 8.5 years) and had less work experience (5.4 ± 8.9 vs. 11.1 ± 7.4 years). Patient satisfaction with the CM consultation was relatively high on a scale from 0 to 24 and was comparable in the two groups (training group: 19.4 ± 4.6; control group 20.5 ± 4.1). The qualitative findings showed that physicians structured majority of consultations as taught during the training. Comparing only the younger and less CM experienced physicians, those trained in CM communication felt more confident discussing CM-related topics than those without training.

**Conclusion:**

A CM communication-training program might be especially beneficial for physicians with less consulting experience when communicating about CM-related issues. A larger trial using more suitable quantitative outcomes needs to confirm this.

**Trial registration:**

ClinicalTrials.gov: NCT02223091, date of registration: 7 February 2014.

## Key message

A complementary medicine communication-training program might be especially beneficial for younger physicians with less experience in communicating with cancer patients about complementary medicine.

## Background

Half of the cancer patients use CM [1]. The European Society of Breast Cancer Specialists has recognized CM as relevant topic [2] and it is increasingly integrated into cancer care as integrative medicine. Clinical practice guidelines for integrative medicine have been drawn up [3]. Although breast cancer patients wish to receive information about CM from the specialist they consult [4, 5], it is rarely a topic of patient-physician communication [4, 6] with specialists being less often informed by their patients about CM use than general practitioners (GPs) [4]. Reasons for not disclosing CM use include a lack of confidence in the physicians’ openness towards the topic, a lack of trust in physician’s CM expertise, and a perception that there is insufficient time to discuss it [4, 7, 8]. Consequently, patients often seek information outside the medical system with an increasing risk of interactions between herbs and anticancer therapies [9]. Physicians often feel uncomfortable discussing CM-related questions and seek training on CM [10]. Besides presenting evidence-based information on CM, guidelines highlight the importance of teaching communication skills [11], since effective communication can positively affect outcomes such as patient satisfaction and quality of life [12, 13]. Although first CM-related trainings are available, a training that focusses on communicating CM and uses a blended learning approach is still missing. This study was part of a collaborative research project, Competence Network Complementary Medicine in Oncology – KOKON, whose aim was to improve evidence-based knowledge on CM among patients and health professionals. The aim was to develop and evaluate a blended-learning training program for physicians for communicating with breast cancer patients about CM. Its feasibility and preliminary effects were evaluated by using quantitative and qualitative research methods (mixed-method approach).

## Methods

### Design

**Fig. 1:**
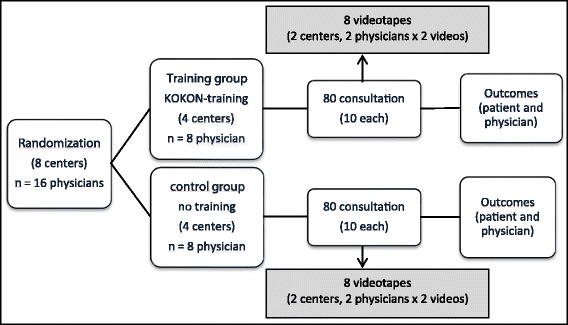
Study design

In a prospective multicenter cluster-randomized pragmatic mixed-method pilot trial we compared a group of trained physicians with an untrained group (control). The study design has been developed with a stakeholder group using Delphi methods (one meeting plus written rounds) including patient representatives, oncologists, psychologists, an expert in media science, an ethicist, GPs and CM experts. A total of eight breast cancer centers or Comprehensive Cancer Centers (CCC) with two physicians each were randomized (Fig. 1). Ten consultations per physician (160 patients in total) were planned. Each consultation was assessed quantitatively by physicians and patients. For the qualitative analysis 16 consultations (eight in each group) were planned to be videotaped and focus group discussions with physicians of both groups were conducted. The study followed the usual guidelines for clinical trials (Declaration of Helsinki and ICH-GCP where appropriate), was approved by the respective Ethics Committees (approval numbers see declaration section) and was registered at ClinicalTrials.gov: NCT02223091. The directors of the participating centers, the physicians and the patients gave written consent to participate in the study.

### Participants

Breast Centers or Comprehensive Cancer Centers in which two physicians were interested in the training were eligible. Inclusion criteria for physicians were: possibility to conduct consultations during working hours, possibility to take part in the on-site skills training in Berlin, not being the physician in charge of the consulted patients and informed consent. Patients were recruited by each of the participating centers by means of information materials or direct patient-physician contact. Patients were eligible if they met the following criteria: age ≥18 years, female, diagnosed with breast cancer, being a patient in the participating center, good German language skills, informed consent.

### Intervention and control

The eight physicians in the intervention group (four centers) received the blended learning KOKON CM training (for content see Table 1) which was developed based 1) on evidence-based CM communication guidelines [11], 2) the consultation handbook that was systematically developed in KOKON and that includes a theoretical model and real-life examples, and 3) didactical methods used in communication and interaction trainings at the Charité – Universitätsmedizin Berlin. Physicians of the control group conducted the consultations based on prior knowledge and professional experience and received the training after the end of the study.

**Table 1:** Training curriculum

Didactical elements	Main objectives/content	Size
Consultation Handbook • Concept of KOKON consultation • Examples from real life consultations	• Acquaintance with key elements of the KOKON consultation (1. realizing and prioritizing the needs and concerns of the patient, 2. strategies to communicate relevant information, 3. evidence about relevant CM-therapies, 4. summary and perspective at the end of the consultation). • Evidence-based information on nutrition, and physical activity for cancer patients. • Most common symptoms and typical patient concerns.	84 pages
e-learning • Reviews • CAM^a^ summaries in German • Consultation videos	• Description of main CM therapies for breast cancer patients (acupuncture, coenzyme 10, ginseng and yoga). • Description of most common consultation situations: demand for general information on available CM therapies, information on specific CM therapies and on CM therapy as alternative for conventional medicine.	9 h
On-site skills training • Role plays with participants • Exercises with data base • Role plays with simulation patients • CM expert presentations	• Ability to apply KOKON consultation elements in daily practice (Recognize the demand of the patient, prioritize, deal with unclear CM evidence and unserious therapies, further procedure). • Use of KOKONBase (database on CM treatments). • Evidence on hormone therapy, mistletoe and cancer related fatigue.	20 lectures (45 min each)

^a^Complementary and Alternative Medicine for Cancer

### Randomization

Centers were randomized using a simple cluster randomization (centers as clusters with two associated physicians as randomization unit) with an allocation ratio of 1:1. The randomization list was compiled from an independent statistician with the program SAS (version 9.3, SAS Inc. Cary, NC, USA). The centers were allocated in order of the receipt of the informed consent by an independent employee of the Institute for Social Medicine, Epidemiology and Health Economics who was not otherwise involved in the study. The study team had no access to the randomization list.

### Quantitative outcome measurements

We collected the physicians’ socio-demographic and professional data at baseline. After each consultation the physician completed a self-assessment about the consultation (general suitability, empathy, structure, complexity and knowledge transfer).

Patients’ outcome measurements after the consultation used adapted versions of the German Consultation and Relational Empathy questionnaire (CARE) [14], German Rating Scales for the Assessment of Empathic Communication in Medical Interviews (REM) [15], questionnaire on satisfaction with inpatient clinical care [16] and a self-developed scale on knowledge transfer and information. The scale consists of 7 items with 5 response options each. (Scored from 0 to 4, range of score 0 to 28, higher values indicate better comprehensibility and higher relevance of information given during the consultation). Furthermore we collected patients’ socio-demographic data.

### Statistics

Data analysis was conducted by descriptive methods. Continuous variables are presented as means, standard deviations, medians and in parts as ranges, categorical variables as absolute and relative frequencies. Satisfaction, empathy and information scores were each modeled in a mixed-effect regression, with intervention as a fixed effect and the physician as a random effect. These models were than extended to calculate the mean differences among the participating physicians, adjusting for age, experience and position (junior physician, specialist, senior physician). Because this is an exploratory analysis we report estimated means and 95 % confidence intervals.

### Qualitative data collection and analysis

Video recordings were taken within the study, and their analysis was based on grounded theory [17, 18]. A content log for summarizing the communication process and setting the interactions and developing initial coding strategies for analysis was done in a group setting by CH + NM. Content logs were coded and analyzed using MAXQDA.

The focus groups were conducted by a trained moderator who was not otherwise involved in the study and followed a semi-structured interview guideline. The guideline was based on the video analysis and included questions about the training, suggestions for improvement, experience with the consultations, the concept, the expectations of the physician toward the consultation, and the manner in which a lack of information about CM was dealt with during the consultation.

The responses of both focus groups were audio-recorded, transcribed verbatim and analyzed using qualitative content analysis [19] with MAXQDA. The discussion of the control and training group were analyzed separately, after which the results were compared.

## Results

### Quantitative results

#### Baseline characteristic of physicians

Of 54 invited centers, 35 were not interested, 11 did not meet the inclusion criteria and eight participated. Although cluster-randomization was conducted physicians’ baseline characteristics differed, with physicians in the control group being more experienced in CM and older (Table 2).

**Table 2:** Baseline physicians’ characteristics

	Training group (*n* = 8) /			Control group (*n* = 9) mean ± sd^a^/		
	mean ± sd^a^	median	range	mean ± sd^a^	median	range
Number of consultation	10.0 ± 0.0	10	10–10	6.3 ± 4.0	7	1–10
Age	33.4 ± 8.9	30.5	27–55	40.0 ± 8.5	37.0	31–55
Years of professional experience in consultation of cancer patients	5.4 ± 8.9	2.5	0.5–27	11.1 ± 7.4	9.0	4–24
	*n* (%)			*n* (%)		
Female gender	7 (87.5 %)			9 (100 %)		
Medical specialist	2 (25.0 %)			7 (77.8 %)		
Oncologist						
Completed	0 (0 %)			4 (44.4 %)		
Envisaged	2 (25.0 %)			2 (22.2 %)		
Gynecologist						
Completed	2 (25.0 %)			5 (55.6 %)		
Envisaged	5 (62.5 %)			1 (11.1 %)		
Other						
Completed	0 (0 %)			1 (11.1 %)		
Envisaged	0 (0 %)			1 (11.1 %)		
Experience in CM consultation (yes)	2 (25.0 %)			3 (33.3 %)		
Self assessment: “Important to be informed about CM”						
I completely disagree	0 (0 %)			0 (0 %)		
I rather disagree	0 (0 %)			0 (0 %)		
I rather agree	3 (37.5 %)			1 (11.1 %)		
I completely agree	5 (62.5 %)			8 (88.9 %)		
Self assessment: “I feel confident in a conversation about CM”						
I completely disagree	2 (25.0 %)			2 (22.2 %)		
I rather disagree	5 (62.5 %)			4 (44.4 %)		
I rather agree	1 (12.5 %)			2 (22.2 %)		
I completely agree	0 (0 %)			1 (11.1 %)		
Self assessment: “Avoid conversation about CM”						
I completely disagree	1 (12.5 %)			4 (44.4 %)		
I rather disagree	4 (50.0 %)			5 (55.6 %)		
I rather agree	3 (37.5 %)			0 (0 %)		
I completely agree	0 (0 %)			0 (0 %)		
Self-assessment: “I wish patients would deal less with CM”						
I completely disagree	4 (50.0 %)			4 (44.4 %)		
I rather disagree	3 (37.5 %)			4 (44.4 %)		
I rather agree	1 (12.5 %)			1 (11.1 %)		
I completely agree	0 (0 %)			0 (0 %)		

^a^standard deviation

#### Baseline characteristic of patients

Patients in the training group were slightly older (mean age: 52.9 ± 11.7 versus 51.3 ± 13.6 years) and better educated (49 % more than 12 years of school education versus 39 %) compared to those in the control group.

#### Feasibility and primary quantitative results

In total, 137 patients had consultations between September 2014 and February 2015 (median number of consultations: intervention group = 10, control group = 7). In the control group one physician took maternal leave and was replaced. The mean consultation duration was 46.5 ± 26.3 min in the training and 29.4 ± 13.4 min in the control group.

**Fig. 2:**
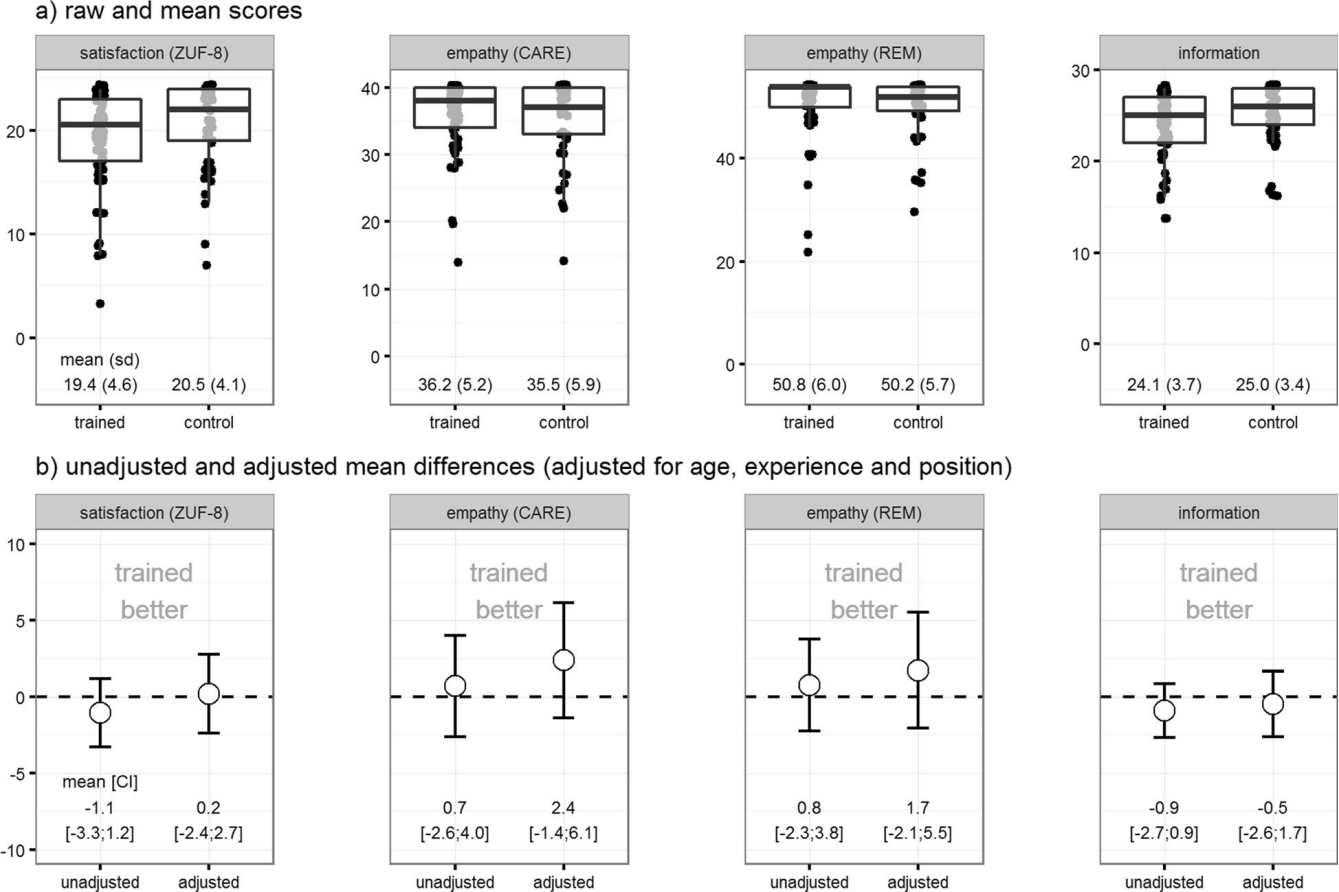
Patients’ assessment of the consultation for satisfaction, empathy and knowledge

Patient satisfaction with the CM consultation was relatively high in both groups (training group: 19.4 ± 4.6; control group 20.5 ± 4.1 on a scale from 0 to 24). After adjustment for age, experience and position the training group had slightly better results for some of the outcomes (Fig. 2). Except one, all the physicians in both groups perceived the consultation setting as suitable and perceived the consultation as positive (Table 3). This one physician did not provide further explanation why the consultation setting wasn’t suitable for him.

**Table 3:** Self-assessment of each consultation by the physicians (6-point numeric rating scale, 1 (*very good/^#^very high), 6 (*not at all/^#^very low))

Item	Training group (*n* = 8) mean ± sd/median /*n* (%)	Control group (*n* = 9) mean ± sd/median /*n* (%)
Number of consultation	80 (58.4 %)	57 (41.6 %)
Overall, consultation situation was suitable (How suitable was the consultation situation to address essential matters?)	79 (98.8 %)	57 (100.0 %)
Empathy (“How well did you succeed in empathizing the patients’ situation and to take this into account during the consultation?)*	1.7 ± 0.5/2.0	1.9 ± 0.7/2.0
Structure (“How well did you succeed in structuring context, content, setting and comprehensiveness of the consultation?)*	2.1 ± 0.8/2.0	1.9 ± 0.8/2.0
Information transfer (“How well did you succeed to impart the information?”)*	2.1 ± 0.9/2.0	2.1 ± 0.7/2.0
Understanding (“How sophisticated was the communication with the patient?”)*	1.6 ± 0.9/1.0	1.6 ± 0.8/1.0
Satisfaction with consultation (“Overall, how satisfied were you with the consultation”) *	2.0 ± 1.0/2.0	2.1 ± 0.8/2.0
Complexity of the consultation (“How complex was the consultation?”)^#^	2.6 ± 1.3/2.0	2.5 ± 1.0/3.0

### Qualitative results

#### Sample description

Fifteen consultations (8 in the training and 7 in the control group, the 8th video consultation in the control group had been cancelled due to maternal leave of the physician) by eight physicians were video-recorded. Three physicians per group considered themselves inexperienced in CM.

#### Characteristics of the consultation sessions

The content of the consultation depended on the stage of the patients’ disease. With newly diagnosed patients the focus was on informing the patients about possible CM treatment options and giving dietary and exercise recommendations. With patients during/after treatment or in the case of metastatic disease consultations targeted specific needs.

The consultations were not only used to discuss CM. The patients also used the time with a physician to address other issues (e.g. anxiety about the cancer itself, concerns regarding chemotherapy).

In the training group the KOKON consultation elements (Table 1) were applied in most of the observed consultations. By contrast, these elements were less often applied by the untrained physicians.

### Focus group discussion

Five of eight physicians from each group participated.

#### Training group

Overall, participants felt comfortable with applying the KOKON consultation structure. They were motivated to conduct high-quality consultations and had the impression that they take more time for consultations than before the training. In particular, physicians in the early stage of their career and who had little experience with CM felt more comfortable consulting on CM and better prepared to deal with gaps in knowledge about CM evidence.

Discussants also expressed interest in further training on CM evidence and in group sessions for a literature review of CM. Some physicians suggested setting up a CM support hotline for complex cases.

Discussants observed that the topics that came up during consultation were not just related to CM; patients often had questions about their conventional therapies. Another point of discussion was the difficulty of integrating CM consultations into clinical routine due to the amount of time needed.

#### Control group

The physicians in the control group felt unprepared for the consultations. This was especially a problem for those at an early stage of their career or with no CM knowledge. To compensate for their lack of knowledge these physicians prepared themselves by searching for information on CM. However, they remained insecure during the consultation.

Physicians found it very satisfying to have a protected time to talk with their patients. Besides CM other questions concerning the disease in general were discussed.

Like their trained colleagues, physicians in the control group, especially those at an early stage of their career, talked about difficulties integrating the consultations into their daily routines.

## Discussion

To our knowledge, this is the first systematic CM communication-training program and the physicians judged it positively. The qualitative results showed that a CM communication-training program might be especially beneficial for physicians with less consulting experience when communicating about CM-related issues.

Many cancer patients wish to be informed about CM by their physicians [4] and the lack of training in the field poses a problem. The combination of various pedagogic elements as provided in our training can enhance satisfaction, learning speed and knowledge of trainees [20, 21]. In addition our curriculum was evidence and experience based.

Patients evaluated the consultation service in both groups very positively and appreciated talking about CM. The consultations provided a setting in which to discuss problems and concerns regarding their disease and treatment effects irrespective of CM.

With regard to quantitative outcomes there were no differences between the groups. Ceiling effects of the outcomes and longer consultation experience of the physicians in the control group might explain these results and more suitable outcomes should be considered in future trials.

However, the training might be especially beneficial for physicians who are young and have less CM experience. Those reported in the focus groups that they felt more secure after the training. This provides information for the ongoing discussion of which group of physicians would benefit most from such a training [22].

Physician in the training group took more time for their consultations compared to those in the control group, most likely because the scope of the consultation was broader for the training group and those physicians gave more consideration to their patient’s situation.

Some physicians obtained information on CM on their own in order to prepare for their consultations. Although information is available on the internet this did not help them to overcome feelings of insecurity when consulting on CM. This highlights the importance of communication skills beyond simple having facts to share in CM consultations, e.g. to understand the patient’s individual motivation about CM treatments.

Beside organizational reasons, insecurity in conducting consultations about CM might have resulted in fewer consultations being held in the control group compared to those in the training group.

The strength of the current study was the mixed-method approach, which provided us with a broad view of the preliminary effects of CM communication training, its acceptance and its implementation into clinical routine care.

Communication skills training in oncology showed small effects for interventions lasting <3 days [22]. Although we tried to balance the shorter duration of the on-site skills training with blended learning elements prolonged on-site skill training might have resulted in larger effects. This was in line with the participants desire to have a longer on-site skills training, but might not be feasible for most clinicians.

The main limitation of the study is that despite cluster randomization the physicians of the control group were older and more experienced in consulting oncological patients. Especially when the number of clusters is small – as it is the case in pilot trials – an unequal distribution of clusters can occur [23]. Randomization at the patient level might have prevented unequal distribution, but this is not practical for a training program as an intervention, because knowledge exchange among the physicians in one hospital is highly probable, and might influence the results.

The participating physicians found it difficult to integrate the CM consultations into their daily routine as the consultations were time-intensive. Moreover, the training was directed to inform patients about CM in an independent consultation with a physician who is not in charge of the patient. A training program that focused on informing a physician’s own patients about CM might be less time-consuming and easier to implement into daily routine.

## Conclusion

Our results suggest that patients appreciated consultations about CM. Young and inexperienced physicians might derive a greater benefit from training on communicating with patients about CM, because their more experienced colleagues may have gained CM knowledge independently during their career. For future studies it is important to develop appropriate outcome measures and to assure comparability of groups.

## Abbreviations

CAM: Complementary and alternative medicine
CM: Complementary medicine
GP: General practitioner
